# Localized Castleman's Disease in the Breast in a Young Woman

**DOI:** 10.1155/2016/8413987

**Published:** 2016-03-17

**Authors:** Rafael Parra-Medina, José Ismael Guio, Patricia López-Correa

**Affiliations:** ^1^Fundación Universitaria de Ciencias de la Salud, Bogotá, Colombia; ^2^Pathology Department, Hospital Infantil Universitario de San José, Bogotá, Colombia; ^3^Mastology Department, Hospital Infantil Universitario de San José, Bogotá, Colombia

## Abstract

Castleman's disease (CD) is a rare lymphoproliferative disorder of unknown etiology. It typically occurs in adulthood but it may also develop in childhood. Clinically, this disease may be classified as localized (unicentric) or systemic (multicentric). Six cases of breast CD have been described in the literature, and all have been reported in adults. Herein we describe the case of a 15-year-old female who presented with a slow-growing tumor in the right breast. The tumor was excised and histopathological examination demonstrated hyaline vascular variant CD. After two years of follow-up, the patient was asymptomatic without evidence of cervical or axillary lymphadenopathy.

## 1. Introduction

Castleman's disease (CD), also known as giant angiofollicular lymphoid hyperplasia, is an unusual condition of unknown etiology. Clinically, it may be classified as localized (unicentric) or systemic (multicentric). Localized CD accounts for most of the cases and presents as a mass located in the thorax (30%), the neck (23%), the abdomen (20%), or the retroperitoneum (17%). It rarely affects the axillary region (5%), the groin area (3%), or pelvic region (2%) [1].

Breast CD is uncommon, and only a few cases have been described in the medical literature [2–6]. This disease most typically develops in patients in their fifth decade. Herein we present the sixth case of breast CD reported in the medical literature, in a young female patient.

## 2. Case Presentation

We present a case of a 15-year-old girl who is 13-week pregnant and consulted the breast specialist due to a 12-month history of noticing a mass in the right breast. It was a slow-growing tumor with no other symptoms. The breast ultrasound scan showed a homogeneous hypoechoic mass with well-defined rounded contours and connective tissue septa showing no calcifications or adjacent tissue retractions. The longitudinal axis, which was parallel to the skin, measured 39 × 36 × 19 mm in size. No other alterations were evidenced. A trucut biopsy of the lesion showed a mature lymphoid infiltrate, possibly of reactive origin, that effaced the ductal structures. Neither necrosis nor mitosis was observed.

**Figure 1:**
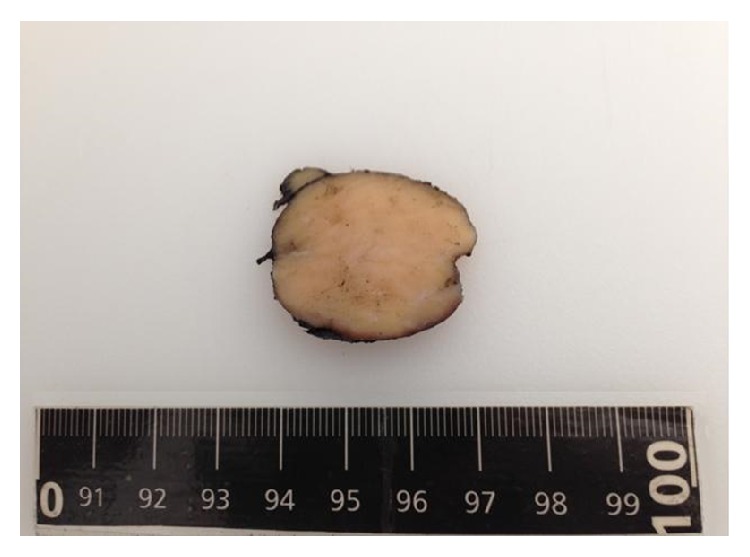
Solid, well-circumscribed tumor measuring 5 × 3.5 × 2.5 cm in size.

The resected quadrantectomy specimen consisted of a mobile tumor, attached to deep structures, with regular margins, firm in consistency and measuring 5 × 3 cm in size. The gross study evidenced a nodular, well-circumscribed, and firm mass, 5 × 3.5 × 2.5 cm in size. Cut sections showed a homogeneous, whitish trabecular pattern with no necrosis on its surface (Figure 1).

**Figure 2:**
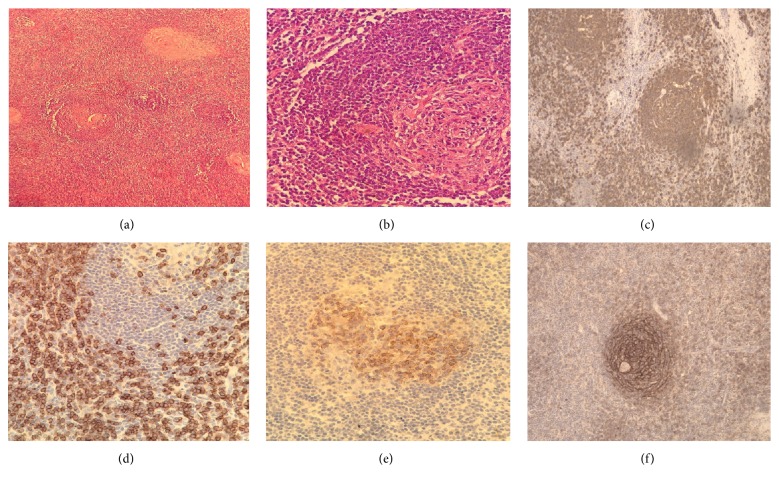
(a) Histology of the tissue showed proliferation of lymphoid follicles (H&E, 20x). (b) Lymph node with lymphoid cells in an “onion skin” pattern with a hyaline center (H&E, 40x). (c–f) Immunohistochemical staining for CD20, CD3, CD10, and CD21 positivity demonstrated follicular hyperplasia.

Histopathology examination demonstrated a typical lymph node background, a capsule, and a remarkably high number of lymphoid follicles. Some of these follicles exhibited atrophic germinal centers, abundant hyaline material, and peripheral wide zones of small lymphocytes surrounding the germinal centers resulting in an “onion skin” appearance, which is a typical feature of CD, as well as hypervascular interfollicular lymphoid tissue displaying numerous proliferative small caliber blood vessels and some obliterated sinuses. Some skeletal muscle fibers within the capsule periphery were also identified. None of the residual mammary tissue was evidenced (Figure 2).

Immunoperoxidase staining showed CD20 reactivity in the B lymphocytes population, CD3 and CD5 reactivity in the T lymphocyte population, CD10, BCL-6, and Ki 67 reactivity in the germinal center population, and CD21 and CD23 reactivity in the follicular dendritic cells. Negativity for HHV8, CD56, TDT, BCL-2, and cyclin D1 markers was also observed. Based on these histological and immune profile findings, the diagnosis of hyaline vascular variant CD was made.

The neck and total abdominal ultrasound scan demonstrated absence of enlarged lymph nodes, which helped to rule out a multicentric disease. At two years' follow-up the patient was asymptomatic and free of cervical or axillary lymphadenopathy.

## 3. Discussion

CD is an uncommon benign condition. It was first described in 1956 by Dr. Benjamin Castleman [7] in a 42-year-old patient with an asymptomatic large mediastinal tumor. To present day, the pathogenesis and etiology of this disease remain uncertain, but a dysregulation in IL-6 overproduction has been described in the unicentric form. Also, an IL-6 alteration associated with human herpesvirus 8 (HHV8) and HIV infection [8] is observed in the multicentric form.

Clinically, the disease may present as a localized form (unicentric) or as a systemic form (multicentric). Keller et al. [9] classified three CD histological subtypes: hyaline vascular type (80–90%), plasma cells type (10%), and mixed type (2%). The hyaline vascular type is the most frequent type and generally presents as a localized form, as a tumor confined to a single lymph node or a group of lymph nodes. Most patients are asymptomatic, but when symptoms are present they are due to compression of neighbor structures related to the size of the tumor. Systemic symptoms are unusual and in many cases incidentally diagnosed [1, 8, 10]. Histology is characterized by an increased number of hyaline small vessels between follicles with obliterated medullary sinuses.

The other two types of CD develop primarily as a systemic form. Histology of the plasma cell type shows a lymph node with preserved architecture and hyperplastic, variable in size lymphoid follicles exhibiting a group of plasma cells between the preserved follicles and sinuses [1, 8, 10].

Even though CD is not considered a malignant disease, it has been associated with an increased risk of developing malignant diseases, such as large B cell lymphoma, POEMS syndrome, follicular dendritic cell sarcoma, paraneoplastic pemphigus, and Kaposi's sarcoma. The latter may be diagnosed simultaneously or after the diagnosis of systemic CD, for these two conditions share the same viral pathogenesis [8].

The diagnosis of CD is based on clinical and histopathology criteria. Differential histopathological diagnoses include toxoplasma lymphadenitis, follicular hyperplasia, follicular lymphoma, HIV lymphadenopathy, mantle cell lymphoma, progressive germinal center transformation, and plasmacytoma [8, 10].

Six cases of breast CD have been described in the medical literature [2–6]. Most cases report an age of onset around or after the fifth decade of life. The size of the tumor varies from 8 mm to 40 mm. Physical examination has reported a mobile mass clinically related to intramammary lymph nodes (ILN) or to interpectoral lymph nodes (Rotter nodes). Histopathologically, the hyaline vascular type is the most common. Just one case has been reported as plasma cell type [3]. A patient developed contralateral infiltrating canalicular carcinoma one year after a CD diagnosis [5] and another patient evolved from a localized disease to a progressive disease and at 4.6 years' follow-up she developed a Hodgkin lymphoma [6]. These lesions in the mammary gland can also be confused with interpectoral lesions [11] or axillary lymph nodes mimicking a breast metastasis [12].

The treatment of localized CD is surgical resection. Patients recover completely (100%) showing a low recurrence rate. The use of systemic therapy with antiretrovirals, glucocorticoids, and biologic therapy [8] is required to treat the systemic form of the disease.

Clinical features, imaging findings, and prognosis of breast CD are not yet well defined due to the lack of case reports on this topic. Thus, publishing more case reports on this condition is required in order to increase knowledge about the natural history of this disease.
